# Extracellular vesicles and exosome-like nanovesicles as pioneering oral drug delivery systems

**DOI:** 10.3389/fbioe.2023.1307878

**Published:** 2024-01-08

**Authors:** Jagannath Mondal, Shameer Pillarisetti, Vijayabhaskarreddy Junnuthula, Sachin S. Surwase, Seung Rim Hwang, In-Kyu Park, Yong-kyu Lee

**Affiliations:** ^1^ Department of Green Bioengineering, Korea National University of Transportation, Chungju, Republic of Korea; ^2^ Department of Biomedical Sciences and Biomedical Science Graduate Program (BMSGP), Chonnam National University Medical School, Gwangju, Republic of Korea; ^3^ Drug Research Program, Faculty of Pharmacy, University of Helsinki, Helsinki, Finland; ^4^ Department of Chemical and Biomolecular Engineering, Korea Advanced Institute of Science and Technology (KAIST), Daejeon, Republic of Korea; ^5^ College of Pharmacy, Chosun University, Gwangju, Republic of Korea; ^6^ Department of Chemical and Biological Engineering, Korea National University of Transportation, Chungju, Republic of Korea

**Keywords:** extracellular vesicles, milk exosomes, exosome-like nanovesicles (ELNVs), oral administration, drug delivery, therapeutic applications

## Abstract

As extracellular vesicle (EV)-based nanotechnology has developed rapidly, it has made unprecedented opportunities for nanomedicine possible. EVs and exosome-like nanovesicles (ELNVs) are natural nanocarriers with unique structural, compositional, and morphological characteristics that provide excellent physical, chemical, and biochemical properties. In this literature, we examine the characteristics of EVs, including how they are administered orally and their therapeutic activity. According to the current examples of EVs and ELNVs for oral delivery, milk and plant EVs can exert therapeutic effects through their protein, nucleic acid, and lipid components. Furthermore, several methods for loading drugs into exosomes and targeting exosomes have been employed to investigate their therapeutic capability. Moreover, we discuss EVs as potential drug carriers and the potential role of ELNVs for disease prevention and treatment or as potential drug carriers in the future. In conclusion, the issues associated with the development of EVs and ELNVs from sources such as milk and plants, as well as concerns with standardized applications of these EVs, are discussed.

## Graphical Abstract

**Figure:**
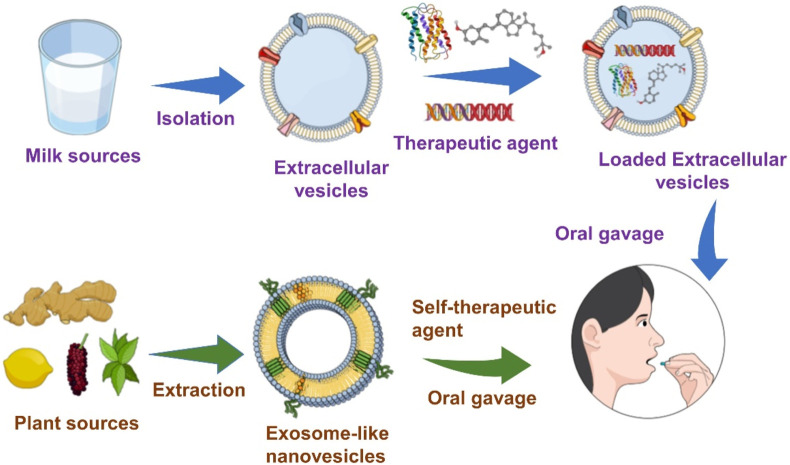
Schematic Diagram: Extracellular vesicles and Exosome-like Nanovesicles (ELNs) are isolated from milk and plant sources and administered via oral delivery for therapeutic applications.

## 1 Introduction

Extracellular vesicles (EVs) are considered pivotal messengers in cell-to-cell communication. EV can transfer endogenous proteins/nucleic acids, such as miRNAs and mRNAs, to recipient cells (Valadi et al., 2007). EVs contain nucleic acids derived from their parental cells, such as specific mRNAs, regulatory microRNAs, other noncoding RNAs, genetic DNA, lipids, and proteins (Hood, 2016; Mondal et al., 2023), and they can transfer endogenous proteins and nucleic acids to recipient cells (Valadi et al., 2007). Although EVs were initially thought to be cell debris, they were later recognized as essential mediators facilitating intercellular transmission of the immune response (Greening et al., 2015), signal transduction (Gangoda et al., 2015), and antigen presentation (Mittelbrunn et al., 2011; Shao et al., 2018). The phytochemical properties of plant EV (PE) and mammalian EV (ME) are very similar (Kim et al., 2021; Lee et al., 2022). A surface charge of −15 mV was applied to PE with hydrodynamic diameters ranging from 50 to 500 nm. PE displayed spherical morphology and a homogeneous structure when examined by TEM or SEM. The PE characterization was similar to the ME techniques mentioned in our previously published article (Mondal et al., 2023). According to Li et al. (2018), by reducing the amount of contaminating protein in the samples, a significantly high particle count to protein concentration ratio can be achieved if the isolation preparation is pure (Webber and Clayton, 2013).

### 1.1 The biogenesis and composition of EVs and exosome-like nanovesicles (ELNVs)

EVs are heterogeneous in types and are classified by their mode of release, their size, originated cells, morphological characteristics, and distinctive functions (Gholizadeh et al., 2017). Small EVs or Exosomes range in size from 30 to 150 nm, while micro-vesicles (MVs) range from 50 to 10,000 nm, and apoptotic bodies (0.8–5 cm) constitute the main subsets, each of which has properties and functions specific to it (Hadizadeh et al., 2022). For instance, apoptotic bodies are produced by cells that are involved in programmed cell death (Yáñez-Mó et al., 2015). MVs can be released from donor cells via shedding known as ectosomes, which are outward budding plasma membranes (Raposo and Stoorvogel, 2013). Alternatively, exosomes are released by fusion of the outer membrane of the multi-vascular bodies (MVB) with the plasma membrane as a result of inward budding of the endosomal membrane (Moldovan et al., 2013). Small EVs, also known as exosomes, are one of the most fascinating subtypes of EVs. EVs usually contain phosphatidic acid, phosphatidylserine (PS), sphingomyelin (SM), cholesterol, and arachidonic acid, which contribute to stability and structural rigidity (Stremersch et al., 2016). EVs are found in various body fluids, including human and animal milk, with substantial variations in EV content between species. For instance, human and bovine milk contains approximately 2.2 × 10^11^ and 1.4 × 10^14^ EVs/mL, respectively (Blans et al., 2017). The proposed origin of milk EVs in the mammary gland is supported by observations of epithelial cell prevalence in human milk, similar microRNA profiles in lactating mammary glands and bovine milk, and the secretion of EV with comparable size and morphology. While the mechanism of ELNVs formation in plants has not been fully elucidated, available data suggest a similarity to mammalian EV biogenesis. Invagination of the plasma membrane by early endosomes initiates EV formation, which requires regulation components of the Golgi trans-network (Hessvik and Llorente, 2018). As an early endosome matures, it invaginates the membrane, forms an intraluminal *vesicles* (ILV), and selectively accumulates various intracellular biomolecules. A mammal’s endosomal sorting complex required for transport (ESCRT) consists of ESCRT-0, ESCRT-I, ESCRT-II, and ESCRT-III proteins, as well as VPS4 ATPase. Multivesicular bodies (MVBs) develop in the late stages of endosome development (Subha et al., 2023). Plants possess most ESCRT proteins and homologs of VPS4/SKD1, suggesting that both proteins may play a role in membrane modifications and endosomal trafficking (Shkryl et al., 2022). The membrane of PEs is composed of digalactosyldiacylglycerol (DGDG), phosphatidic acid (PA), monogalactosyldiacylglycerol (MGDG), and phosphatidylcholines (PCs) (Teng et al., 2018). Thus, the variations in lipid characteristics provide essential mammalian cell-regulating functions. Among phospholipids, one of the intriguing properties of PA is that it targets and activates the mammalian target of rapamycin (mTOR), commonly found in PEs. The mTOR pathway activates several human health and disease processes, including cell growth, proliferation, and recovery. Compared to MEs or synthetic nanoparticles, PEs are believed to have more therapeutic effects because of their advantageous characteristics. These benefits include ease of bulk-scale production (Li et al., 2018), low toxicity, low immunogenicity (Deng et al., 2017), effective cellular absorption (Wang et al., 2013), and good biocompatibility and stability (Zhang et al., 2016a).

With their significant physicochemical advantages, EVs and ELNVs have been considered intriguing candidates for therapeutic applications. The most crucial limitation of sources of drug delivery systems with adequate bioavailability has led to significant challenges to scientific communities. Interestingly, researchers have found that the sources of edible foods could address the limitations of sources of drug delivery systems. Edible foods, such as mammalian milk, plants, and mammalian exerted materials, such as saliva, semen, and urine, have been investigated for biomedical applications (Doyle and Wang, 2019; Zhong et al., 2021; Kim et al., 2022). Mammalian milk (human breast milk, bovine, porcine, cow) and plants (root, fruit) are considered possible sources of EVs and ELNVs for oral delivery. With outstanding achievements in sophisticated nanobiotechnology, EVs, and ELNVs have been investigated extensively for therapeutic effects. Recently, scientists have extended the use of EVs and ELNVs for oral delivery systems due to their physiological stability in gastrointestinal fluids (Zhang et al., 2017; Wang et al., 2019; Wu et al., 2022). This review aims to highlight EVs and ELNVs for up-to-date therapeutic applications via oral delivery and their advancements toward clinical translation.

### 1.2 Mechanism for loading drugs into EVs and ELNVs

EV composition in plant cells is regulated by a molecular mechanism that has long been unanswered. Currently, most studies focus on native EVs from plants containing biomolecules such as proteins, miRNAs, and species-specific secondary metabolites (Dad et al., 2021)7. ELNVs’ therapeutic potential depends heavily on the species of plants used for isolation (Hessvik and Llorente, 2018).

It has been shown that milk EVs have considerable potential as drug delivery vehicles (Tian et al., 2023). There are two main methods for loading into nanovesicles: active and passive. In active loading, the membrane of the nanovesicle is briefly disrupted by techniques such as sonication, extrusion, and freeze-thaw cycles, allowing the inward diffusion of various compounds, and then the membrane is reassembled (Fuhrmann et al., 2015). On the other hand, passive loading occurs through an incubation process, where loading takes place (Vashisht et al., 2017). This incubation can be carried out through two methods: incubating nanovesicles with a compound or incubating them with donor cells containing the target molecules (Zhang et al., 2016a).

## 2 EVs as delivery vehicles

EVs are considered potential delivery systems due to their physiochemical advantages. Both hydrophobic and exogenous hydrophilic or lipophilic agents are encapsulated into EVs. Several studies have demonstrated that EVs have been used to deliver endogenous proteins, RNAs, DNA, *etc.*, to treat various disease-specific diseases. For example, short interfering (si)RNAs (siRNAs) were encapsulated into dendritic cell (DC)-derived EVs in the mouse brain by systemic injection of targeted EVs. EVs from DCs were explored to escape immunogenicity and modified with RVG peptides for tissue-specific targeting. Exogenous siRNAs were loaded into EVs by electroporation. The results demonstrated specific gene (mRNA) (60%) and protein (62%) knockdown of BACE1 compared to other tissues. This study showed that the developed targeted EVs could demonstrate the therapeutic potential of siRNAs in wild-type mice for treating Alzheimer’s disease (Alvarez-Erviti et al., 2011).

Interestingly, Nawaz *et al.* performed a comparative study of lipid nanoparticles (LNPs) and EVs in transporting VEGF-A mRNA to heart-specific cell lines to evaluate blood vessel formation. This study suggested that EVs could be potential candidates for the delivery of mRNA to the heart or other organs of interest, which need further investigation for customization. The results showed the detection of VEGF-A mRNA in EVs, indicating that LNP-VEGF-A mRNA was taken up by the cells and secreted to VEGF-A mRNA-loaded EVs later. Overall, this study demonstrated the effective delivery of VEGF-A mRNA for angiogenesis and could be investigated to customize specific cells/tissues for mRNA delivery (Nawaz et al., 2023).

Moreover, the surface of EVs is modified with proteins, peptides, antibodies, or specific targeting moieties to enhance the targeting ability of delivery systems. In addition, genetically modified parental cells were utilized to produce EVs and their various treatments. Hybrid EVs have been developed for effective therapeutic outcomes to maintain the integrity of biocompatibility and improve stability. We have briefly discussed EVs and the advancement of EVs as delivery vehicles in recently published literature (Mondal et al., 2023).

## 3 EVs as oral delivery vehicles

Low pH and degradative enzymes in the stomach limit sufficient drug absorption in the gastrointestinal tract (GI). To circumvent these difficulties, intravenous administration was considered for patient treatment. These intravenous infusion techniques are associated with reduced sterility, inconvenience, and poor cost-effectiveness. Therefore, oral delivery systems have been considered an alternative, patient-friendly strategy. Here, EVs derived from mesenchymal stem cells have been administered via intravenous injection and improved ulcerative colitis (UC) to some extent. Chao and coworkers proposed layer-by-layer (LbL)-coated EVs (LbL-Exos) for treating UC oral delivery to overcome this issue. EVs were coated with N-(2-hydroxyl) propyl-3-trimethyl ammonium chitosan chloride (HTCC) and oxidized konjac glucomannan (OKGM) polysaccharides to target the colon as well as protect against degradation in the GI tract. The study showed that LbL-Exo groups significantly improved body weight, colon length, and disease activity index (DAI) score compared to Exos IV, Exos oral, LbL oral, or other experimental groups.

Moreover, oral treatment with LbL-Exos drastically improved inflammation in DSS-induced colitis compared to other groups. Common proinflammatory cytokines, such as IL-6 and TNF-α, were highly detected in DSS-treated mice’s serum and colon tissues treated with 5-ASA, iv. Exos or oral LbL-Exos significantly reduced these cytokines. In addition, the percentage of F4/80+CD86-labeled M1 macrophages decreased in DSS-treated mice treated with 5-ASA, i.v. MSCs, iv. Exos and oral. LbL-Exo treatment compared to the control group (PBS). Meanwhile, F4/80+CD206+-labeled M2 macrophages increased 4-fold with the treatment of LbL-Exos compared to the PBS group. In conclusion, the developed LbL-Exo system exhibited significant improvement in anti-inflammation and tissue repair ability and may pave the way for verse applications for oral delivery in various GI diseases (Deng et al., 2023). However, the oral administration of many therapeutic biologics continues to be challenging. EVs or ELNVs extracted from sources such as milk or edible plants may pass through the intestinal barrier and be considered potential oral medication vectors (Mu et al., 2014; Zhang et al., 2016a; Lin et al., 2020).

### 3.1 Milk EVs as an oral delivery systems

EVs from milk are absorbed in the GI tract (Liao et al., 2017; Betker et al., 2019) and remain stable (Jiang et al., 2021) in harsh environments, which could be an excellent achievement for oral drug delivery vehicles. However, issues associated with producibility and affordability demand further development or investigation (Chen et al., 2022). Recent findings demonstrated that EVs from milk were auspicious candidates for oral administration to resolve the aforementioned limitations (Aqil et al., 2017; Lin et al., 2020; Wu et al., 2022). Therefore, EVs from milk have been considered a potential vehicle for encapsulating and delivering biotherapeutic macromolecules (Adriano et al., 2021; del Pozo-Acebo et al., 2021; Zhong et al., 2021). Many studies have documented that the neonatal Fc receptor (FcRn) is highly expressed in the GI tract and efficiently binds to IgG (Sockolosky and Szoka, 2015; Martins et al., 2016). Anchordoquy *et al.* previously reported significant quantities of IgG present in EVs from milk (Graner et al., 2009; Hellwinkel et al., 2015). Interestingly, a clinical study showed that miRNAs in EVs extracted from milk were absorbed into the blood of human patients at sufficient levels to express genes in blood cells (Baier et al., 2014; Zempleni et al., 2017).

Therefore, the unique property of GI stability has attracted considerable attention as an oral delivery vehicle for milk EVs. Jamie *et al.* studied the role of FcRn in the gut absorption of EVs derived from cow milk. In addition, they showed the biodistribution of EVs to significant organs (the lung, kidney, heart, spleen, and liver) and the accumulation of EVs at the tumor site (Anchordoquy, 2019).

#### 3.1.1 EVs as gene carriers

The primary and essential nutrient source of newborn mammals is breast milk. miRNAs play a crucial role in immune function (Kosaka et al., 2010), development (Chen et al., 2006), differentiation (Song and Tuan, 2006), proliferation (Sluijter, 2010), and metabolism (Wilfred et al., 2007). miRNA is abundant in milk fat globules (Li et al., 2016), whey (Izumi et al., 2014), and EVs (Huang et al., 2013).

Delin *et al.* assessed miRNA levels in piglet serum by feeding them EVs derived from different species, such as bovine or porcine milk, and the absorption of miRNAs in mammalian infants. The results showed that the miRNA levels varied in piglet serum when piglets were fed bovine or porcine milk EVs. The levels of miR-2284× and miR-2291 were markedly higher after feeding bovine milk, whereas miR-7134 was significantly improved in pigs fed porcine milk compared to those fed bovine milk. This finding facilitated a significant comparative evaluation of the physiological functions of miRNAs in infants by feeding them milk sourced from different species (Lin et al., 2020). This study revealed that mammalian infants can absorb miRNAs from milk sources. Moreover, exosomal milk miRNA derived from buffalo milk was characterized and examined for therapeutic benefit (Chen et al., 2020). Buffalo milk is an abundant source and is the second most highly consumed milk globally (Rani et al., 2020).

The bioavailability and distribution of EVs and their microRNAs derived from different species, such as bovine, porcine, and murine milk, within or across species were assessed by Sonia *et al.* Distinct miRNAs in bovine milk EVs showed different efficacies via oral administration. Milk EVs were shown to deliver protein and RNA cargo to the brain and spleen, liver, or heart. Unfortunately, EVs from bovine milk regretted rescuing Drosha homozygous knockout mice due to insufficient quality and relatively low bioavailability of essential milk miRNAs (Zhong et al., 2021).

Bovine milk was used as an abundant source for the high yield of EV production. EVs from milk are considered nontoxic and tolerable for delivering hydrophilic molecules such as peptides, proteins, and genes. This study demonstrated the stability of milk EVs in the intestinal tract. The stability of EVs and loaded siRNA was confirmed by *in vitro* digestion of EVs from milk, which revealed no significant difference between colocalized yellow color production for digested and undigested EVs from milk. This study significantly showed the potential of bovine EVs as an oral RNA carrier system that can protect cargo from the harsh barrier of the gastrointestinal tract (Shandilya et al., 2017).

Matthew R. Warren demonstrated that highly pure EVs from bovine milk (mExo) with surface modification presented an advanced oral delivery system for siRNA. The mExos were functionalized with polyethylene glycol (PEG), improving gastric environment stability and significantly enhancing cellular uptake by IECs. In addition, the loading efficiency and membrane integrity of mExos were assessed by altering conventional methods, such as cationic chemical transfection and electroporation. The loading efficiency of siRNA into the mExo was higher when using chemical transfection than when using electroporation. Finally, the PEG-coated mExo-loaded siRNA showed a significant gene silencing effect *in vitro*. The authors claimed that the modified mExos needed a low-cost enrichment process, facilitated higher purity, and could be a promising naturally derived vehicle for the oral administration of siRNA (Warren et al., 2021).

**FIGURE 1:**
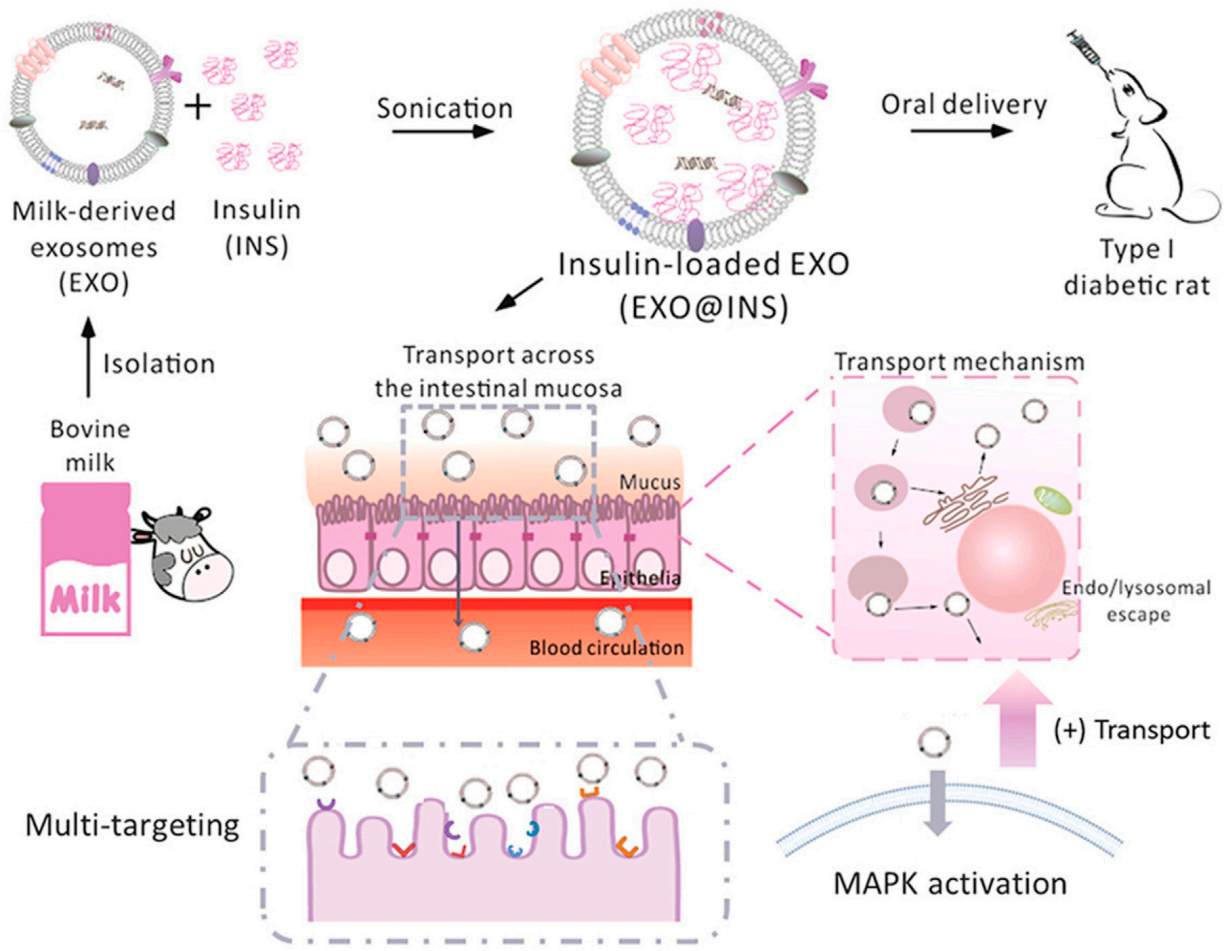
Schematic presentation of the isolation of EXO from bovine milk and loading of peptides (insulin) for oral delivery to mice. Reproduced with permission from Wu et al. (2022). Copyright 2022 Elsevier. Exo, milk-derived exosomes; INS: insulin: EXO@INS: insulin-loaded exosome.

In the case of therapeutic peptide delivery, Wu *et al.* took advantage of the stability of EVs from bovine milk in the GI tract and were able to scale up at a large scale, as milk is more affordable and abundant than cell culture-derived EVs (Wu et al., 2022). Here, the investigation of EVs extracted from milk as drug delivery systems is illustrated in Figure 1. Milk-derived EVs show more excellent stability while passing through the GI tract, including stability at the stomach site. As expected, EVs from milk loaded with insulin were protected, although the GI tract barrier showed better mucus penetration ability. EVs from milk loaded with insulin showed sustainable hypoglycemic effects compared to subcutaneously delivered insulin. This study developed a simple and cost-effective carrier system that can be produced at a large scale. Significantly, Zhang *et al.* developed an oral mRNA vaccine using EVs from bovine milk to prevent SARS-CoV-2 infections. The SARS-CoV-2 receptor-binding domain (RBD) was encapsulated into EVs and tested in 293 cells.

Interestingly, RBD mRNA-loaded Exos effectively produced the RBD peptide after 24 h, consequently neutralizing antibodies against the RBD in mice. This study was a preliminary assessment of developing an oral vaccine using EVs from milk to protect against COVID-19. This study showed an easy and inexpensive strategy to develop mRNA vaccines for oral delivery (Zhang et al., 2023).

#### 3.1.2 EVs as drug carriers

EVs have great potential to act as intelligent carriers for the oral delivery of anticancer agents and macromolecules such as proteins and peptides. Aqil *et al.* took advantage of milk EVs to overcome the chemoresistance of ovarian cancer (Aqil et al., 2017a). It is considered difficult to treat ovarian cancer because of the development of chemotherapy resistance, and new approaches are needed to treat chemoresistant cancer. Berry-derived anthocyanidins (anthos) show great preventive and therapeutic activity against various cancers. In this research, they checked the potential of Anthos against drug-resistant cancer cells. Cisplatin drug treatment in combination with Anthos shows promising anticancer effects. However, Anthos delivery lacks oral bioavailability and stability, which need to be accounted for. Considering this limitation of plant bioactivity (Anthos), this study took advantage of bovine milk-derived EVs to deliver berry Anthos via the oral route. Oral delivery of Anthos showed dose-dependent cancer cell killing and promising anticancer effects in combination with cisplatin. There is often retained sensitivity toward paclitaxel drugs in cisplatin-resistant tumors, and systemic administration of paclitaxel results in severe side effects. To overcome these challenges, an EV-loaded paclitaxel formulation was developed—EVs derived from milk help to deliver plant bioactive and anticancer drugs such as paclitaxel and cisplatin. A synergistic effect was observed by combining two EV-based oral formulations, exosomal Anthos (ExoAnthos) and exosomal paclitaxel (ExoPAC), and a significant anticancer effect was achieved. This study highlighted the potential of milk-derived EVs to deliver plant bioactive compounds, which have poor oral bioavailability.

**FIGURE 2:**
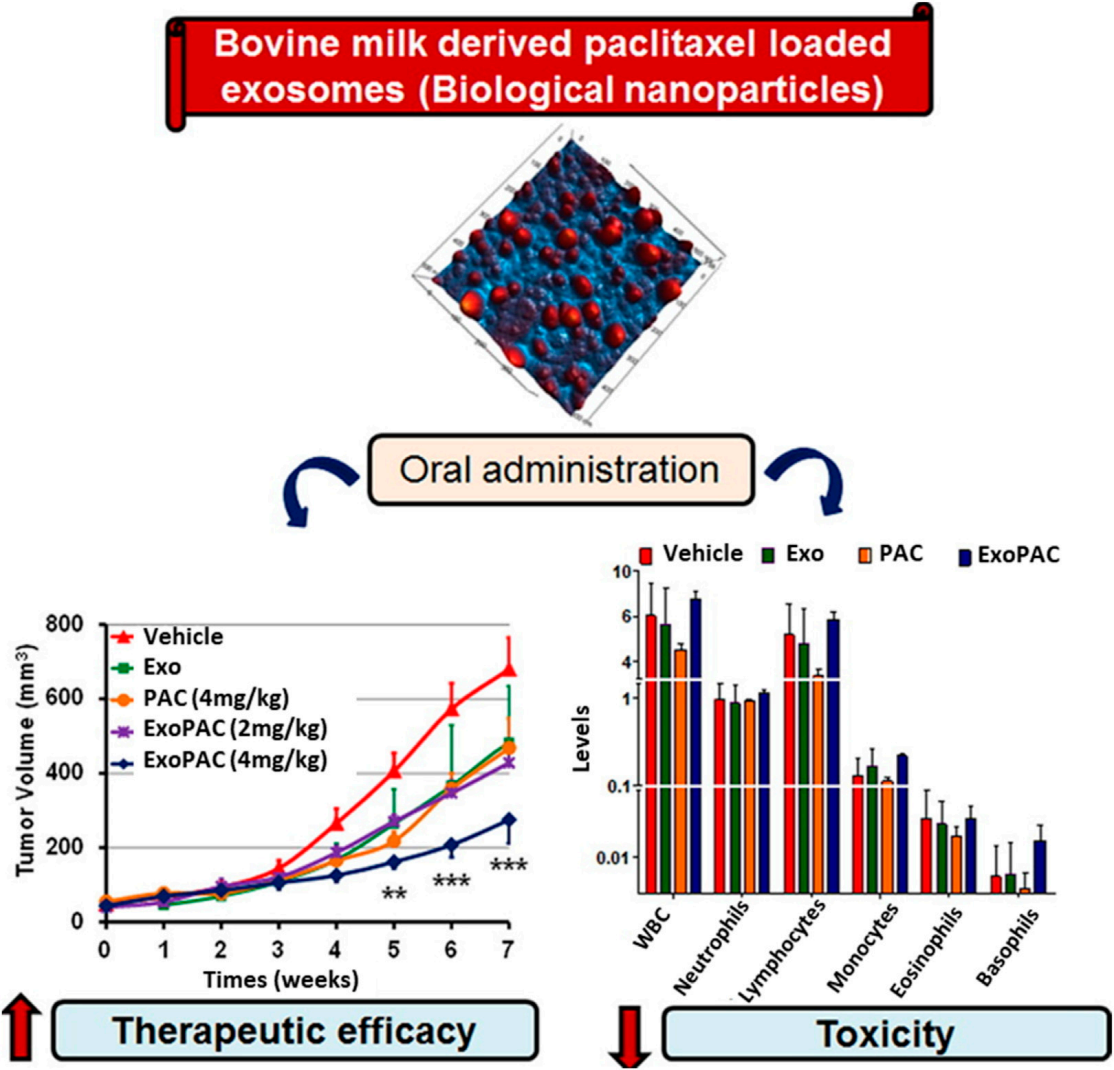
ExoPAC shows significantly increased therapeutic effectiveness and reduced systemic and immunologic toxicity after oral gavage. Reproduced with permission from Agrawal et al. (2017b). Copyright 2017 Elsevier. Exo, exosome; PAC, paclitaxel; ExoPAC, paclitaxel-loaded exosome.

Although PAC has excellent therapeutic effects, it has some limitations, such as poor water solubility and significant toxicity. Agrawal *et al.* introduced PAC drugs for oral delivery as a patient-friendly approach to conventional i.v. Treatment of human lung tumor xenografts mice model to overcome such limitations. EVs from cow milk were utilized as a delivery system with suitable physiological properties, especially their size, uniformity, surface charge, and loading capacity of 8%. PAC-loaded EVs (ExoPAC) and EVs were stable in gastrointestinal fluids under extreme conditions, such as −80°C. During *in vitro* testing at pH 6.8, ExoPAC exhibited sustained release profiles for up to 48 h. In nude mice, ExoPAC was administered orally to treat lung tumor xenografts to evaluate its therapeutic efficacy, inhibiting tumor growth by 60% (p0.001). In addition, systemic and immunogenic toxicities were tested using ExoPAC, demonstrating negligible toxicity compared to intravenous PAC (Figure 2) (Agrawal et al., 2017b).

Later, the same group demonstrated bovine colostrum-derived EVs as carriers for PAC to treat lung cancer via oral administration. In this study, the author reported that EVs produced in higher yield from colostrum and colostrum-derived EVs showed improved loading efficacy compared to milk-derived EVs. Initially, ExoPAC was utilized against A549 cells, and consequently, folic acid was attached to ExoPAC (FA-ExoPAC) to improve its efficacy further. Oral administration of FA-ExoPAC revealed a significant reduction in tumor xenografts (50%) compared to PAC alone. Moreover, they translated this system into an orthotopic lung cancer model to determine whether the study yielded better results. It was found that this treatment produced more significant growth inhibition (55%) than i.v. PAC (24%–32%) and similar results as i.v. Abraxane (59%). Based on its significant improvements in overall efficacy and safety profile, ease of handling, and cost-effectiveness, it could be a potential candidate to treat lung cancer rather than i.v. PAC and Abraxane (Kandimalla et al., 2021).

Curcumin (CUR) is a well-known chemopreventive agent and has been explored in preclinical studies against various diseases, including cancer (Anand et al., 2008). The lack of water solubility of CUR limits its therapeutic application. Several materials, such as polymeric nanoparticles, micelles, solid lipid nanoparticles, liposomes, and other formulations, have been utilized for delivery vehicles. However, these approaches have inherent drawbacks, such as stability issues and short circulation times (Wang et al., 2009). Aqil *et al.* proposed that when administered orally, CUR-loaded exosomes (ExoCUR) ameliorated inflammation and tumor growth. In this study, EVs were extracted from milk, and ExoCUR with improved physiological properties, such as loading efficacy, particle size, stability, and surface properties, were investigated for therapeutic effects.

ExoCUR displayed improved antiproliferative and anti-inflammatory properties against cancer cell lines (lung, breast, and cervical cancer) compared to free CUR. The tumor xenograft model was constructed and administered ExoCUR by oral gavage to evaluate antitumor activity, significantly inhibiting tumor growth by ∼61%. In addition, ExoCUR was bioavailable and showed negligible toxicity (Aqil et al., 2017b). Another group, Vashisht *et al.*, investigated the stability and solubility of curcumin in milk EVs (Vashisht et al., 2017). As highlighted by earlier studies, milk-derived EVs are stable in harsh GI conditions and have great potential to enhance the oral bioavailability of plant bioactive compounds. Curcumin stability and bioavailability were greatly enhanced by delivering curcumin with the help of milk EVs.

**FIGURE 3:**
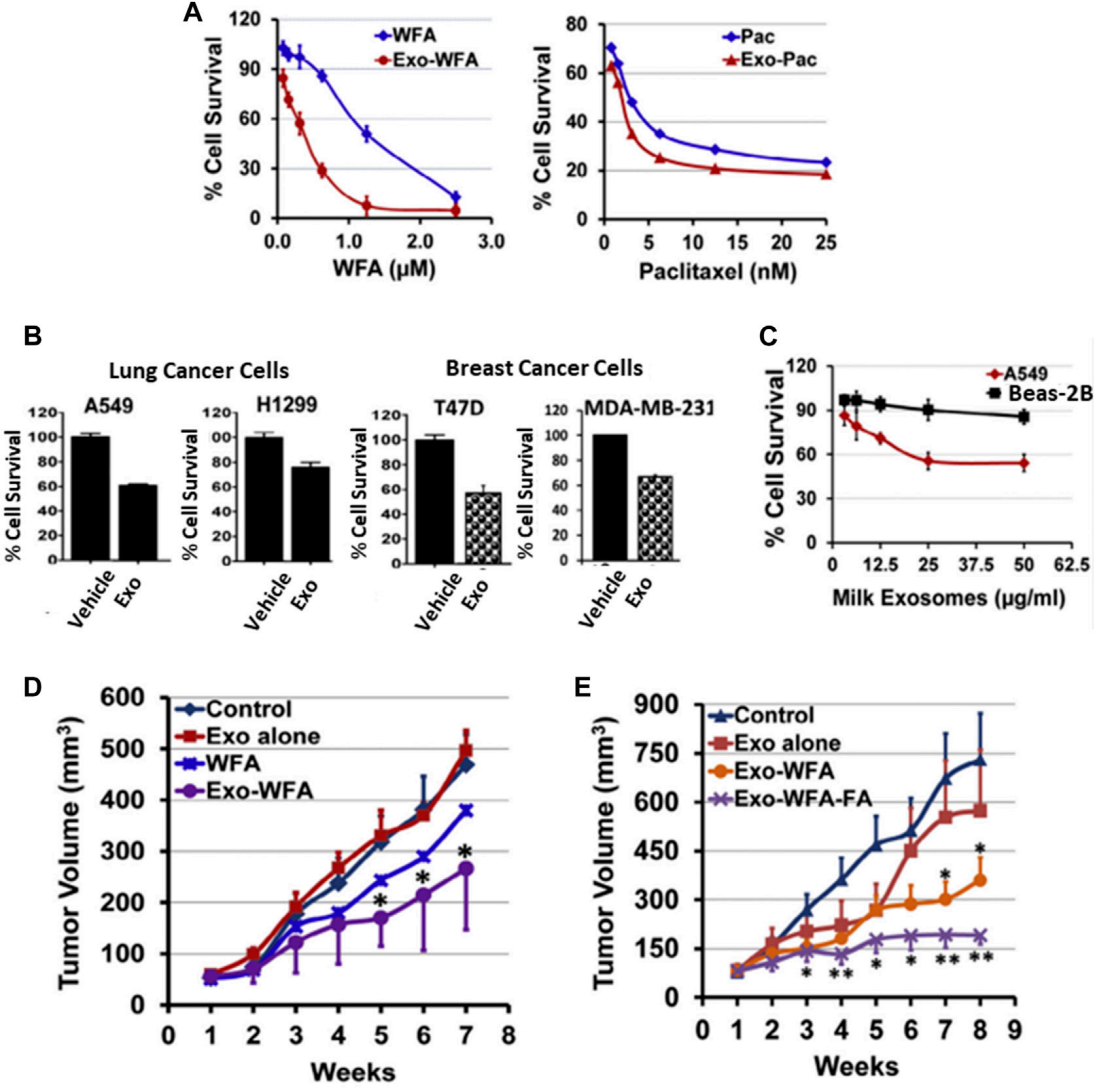
Bovine milk-derived EVs can be used as a DDS and show therapeutic activity. **(A)** Antiproliferative activity of drugs encapsulated in EVs and free drugs. **(B)** Antiproliferative activity of EVs against human lung and breast cancer cells. **(C)** Antiproliferative activity of EVs at 0–50 μg/mL concentrations for 72 h against normal human lung and lung cancer cells. **(D)** Antitumor activities of WFA, Exo, and Exo-WFA by intravenous and oral administration **(E)**. Reproduced with permission from Ref Munagala et al. (2016). Copyright 2016 Elsevier. WFA, Withaferin A; WFA-FA, Withaferin A-folic acid; PAC, Paclitaxel.

Milk from bovine has been considered a scalable source of EVs in terms of safety. Munagala *et al.* recently investigated EVs as carriers of chemotherapeutic/chemopreventive agents. The authors demonstrated that both hydrophilic and hydrophobic small molecules, such as withaferin A (WFA), bilberry-derived anthocyanidins (Anthos), CUR, PAC, and docetaxel (DOC), were encapsulated into EVs for therapeutic investigations. These formulations improved the therapeutic efficacy of oral gavage. Moreover, folic acid (FA)-functionalized drug-loaded EVs showed better effects than others. Therefore, milk-derived EVs could be a fitting candidate for the delivery system, as these are potentially scalable, cost-effective, and biocompatible (Figure 3) (Munagala et al., 2016). In another study, Qu *et al.* demonstrated α-mangostin-loaded EVs (AExos) for antibacterial activities, resulting in 99% clearance of bacteria in macrophages.

Moreover, AExos exhibited proficient solution stability, acid resistance, and high mucus penetration ability for poorly soluble drugs in aqueous solutions. AExo has been tested in two infected animal models, the mouse intestinal infection model and the chicken necrotic enteritis model, to evaluate antibacterial activities by oral delivery treatment. The results demonstrated that the bacterial loads were significantly reduced in the large intestine in the AExo-treated group compared to the LZD- and AMG-treated groups. Furthermore, the relative abundance of the *Lactobacillus* and Lactococcus genera in the chicken cecum was increased after treatment with AExo compared to other groups (Qu et al., 2022). Li *et al.* demonstrated drug loading assessment into EVs from bovine milk for developing promising delivery systems for hydrophilic biomacromolecule drugs. In this study, several methods, such as sonication, saponin, incubation, and freeze/thaw, were used to evaluate the loading efficacy of drugs. In addition, drug lipophilicity and molecular weight accounted for the loading capability. The results demonstrated that hydrophilic drugs were loaded significantly higher than hydrophobic drugs. A more considerable molecular weight of hydrophilic drugs was encapsulated into EVs with greater loading efficiency using sonication and the saponin method, while the loading efficiency of hydrophilic drugs varied. Multiple hydrophilic macromolecules were tested to cross-check the loading and release profile of hydrophilic macromolecules in EVs, and their results were validated. In conclusion, this study demonstrated the development of hydrophilic macromolecule-loaded EVs for oral delivery systems (Li et al., 2023).

A comparative study showed enhanced oral bioavailability between cow milk and IEC-derived EVs. Both EVs were loaded with CUR and checked for cellular uptake and permeability in Caco-2 cells. IEC-derived EVs resulted in slightly better cellular uptake than cow milk-derived EVs. However, both EVs enhanced the cellular uptake and intestinal permeability of CUR, reflecting milk EVs’ potential for improving oral drug bioavailability (Carobolante et al., 2020).

## 4 Role of the source of ELNVs in therapeutic efficacy

**FIGURE 4:**
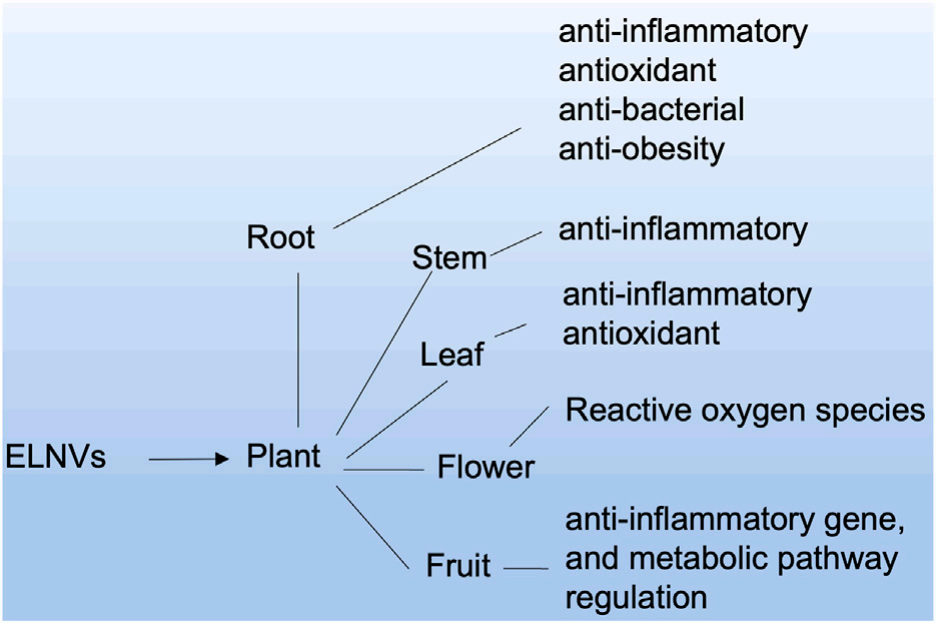
ELNV-derived sources and their potential therapeutic applications.

ELNVs are derived from different cell sources, especially for oral delivery systems. Plant sources are considered potential carriers. Plant roots, seeds, juice, and dried plant materials can be used to isolate EVs (Nemati et al., 2022). ELNVs derived from each source have their credibility toward therapeutic potential (Figure 4). For example, the root source of ELNVs has exhibited anticancer, anti-inflammatory, antioxidant, and anti-obesity properties. However, fruit-derived ELNVs showed anti-inflammatory, gene, and metabolic pathway regulation properties. Other parts of plant-like bark sources exhibited anti-inflammatory effects, and tea plant flowers showed reactive oxygen species production.

### 4.1 Root-derived EVs and ELNs as an oral delivery system

Like mammalian EVs, plants can also release EVs in the extracellular space for communication. Plant-derived EVs are considered more biocompatible, eco-friendly, and easy to mass produce than animal-derived EVs. Exosome-like particles derived from ginger and carrot show anti-inflammatory properties. Several critical applications of plant-derived EVs, such as anticancer and anti-inflammatory effects, and the importance of these particles in maintaining intestinal homeostasis show their potential in biomedical applications. Apart from their therapeutic effects, they can successfully deliver EVs and endogenous agents to animal organs.

**FIGURE 5:**
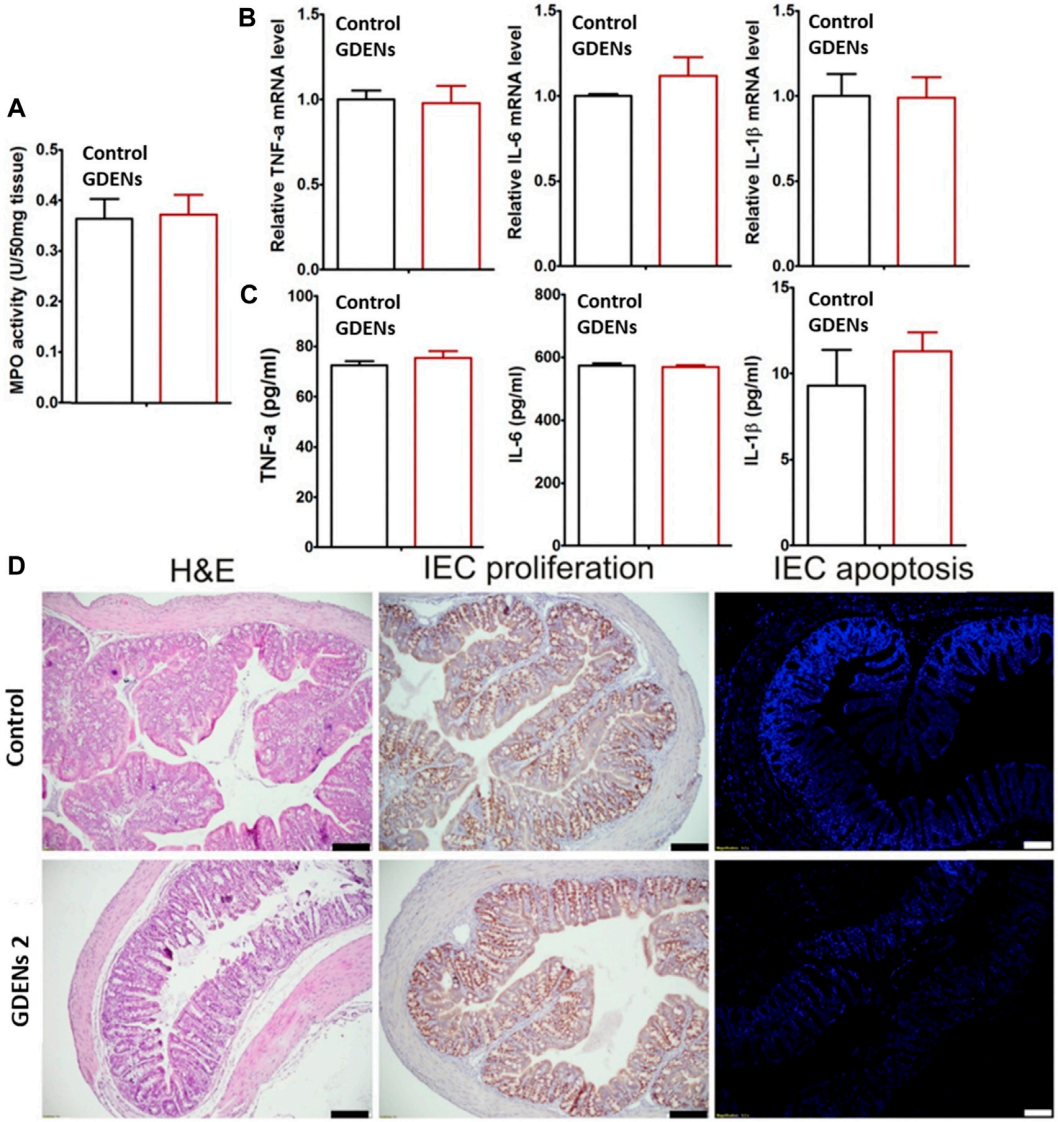
ELNVs derived from ginger performed well in either local or systemic administration when administered by oral gavage compared to the control (without treatment). **(A)** The activity of colonic myeloperoxidase (MPO). **(B,C)** Quantifying the number of proinflammatory cytokines (TNF-α, IL-6, and IL-1β) at the mRNA and protein levels. **(D)** The results of H&E staining of colonic tissues and the proliferation and apoptosis of IECs. The scale bar represents 100 μm. (n = 5). They are reproduced with permission from Zhang et al. (2016a). Copyright 2021 Elsevier—GDENs, ginger-derived ELNVs; MPO, colonic myeloperoxidase.

A novel therapeutic strategy was developed by Zhang *et al.* to prevent and treat colitis-induced inflammatory bowel disease (IBD). Here, edible ginger-derived ELNVs (GDENs) acted as therapeutic agents and were administered orally to treat inflammatory bowel disease and ulcerative colitis. To evaluate the effects toxicity of GDENs, colon-26 epithelial and RAW 264.7 macrophage lines were treated with GDENs. The cell viability is evident, with no change after treatment with GDENs compared to nontreatment. After careful evaluation of GDENs in an *in vitro* study, they translated GDENs into *in vivo* cells to determine whether they had any significant toxicity. To evaluate the cytotoxicity of GDNPs, they were administered orally for 7 days, and the mRNA or protein levels of proinflammatory cytokines, such as TNF-α, IL-6, and IL-1β, and colonic MPO activity were measured. This study confirmed significant toxicity with or without GDEN treatment (without treatment as a control). Moreover, when subjected to H&E staining, no differences in intestinal epithelial cell (IEC) proliferation or IEC apoptosis were observed between the GDEN-treated groups and control groups (Figure 5). After careful toxicity evaluation, mice with dextran sodium sulfate (DSS)-induced colitis were administered with GDENs to assess therapeutic efficacy. An interesting finding was that the reduction in the numbers of proinflammatory cytokines, such as TNF-α, IL-6, and IL-1β, as well as the increase in anti-inflammatory cytokines, such as IL-10, were increased significantly after treatment with GDENs in DSS-induced colitis. This nanovesicle could be a potential therapeutic agent and can also be explored in therapeutic delivery to treat colitis-related diseases (Zhang et al., 2016a). In another attempt, Zhang *et al.* effectively delivered siRNA into GDENs for ulcerative colitis therapy. SiRNA-GDENs had specific targeting properties for colon tissues and decreased CD98 expression in colon tissues. To circumvent the problems associated with nonspecificity and economic burden, GDENs could be considered intriguing carrier alternatives to traditional synthetic nanoparticles for oral DDSs (Zhang et al., 2017). In another study, Zhuang *et al.* reported that GDENs effectively protect against alcohol-induced liver injury by activating nuclear factor erythroid 2-related factor 2 (Nrf2). GDENs demonstrated significant accumulations in hepatocytes compared with other organs by oral gavage. Therefore, GDENs could be implemented as next-generation potential targeting candidates against liver-specific diseases (Zhuang et al., 2015). Interestingly, GDENs were investigated to evaluate the absorption kinetics in the intestine in rats. The study successfully demonstrated a right absorption trend of GDENs (concentration of 15–60 mg/mL) as duodenum > jejunum > ileum (Man et al., 2021).

Mu *et al.* also explored edible plant-derived ELNVs (EPDENs) to investigate their biological activity in mammalian cells. Some of the ELNVs explored from root sources of plants are ginger and carrot. Ginger-derived EVs preferentially induced antioxidative genes such as heme oxygenase-1 (HO-1) and anti-inflammatory cytokines such as IL-10, whereas carrot-derived EVs increased the activation of nuclear factor (erythroid-derived 2)-like-2 (Nrf2) in macrophages. There was no influence of EPDENs on interspecies communication or biological activity. Therefore, EPDENs could be potential delivery vehicles for DDSs because they are naturally abundant (Mu et al., 2014).

**FIGURE 6:**
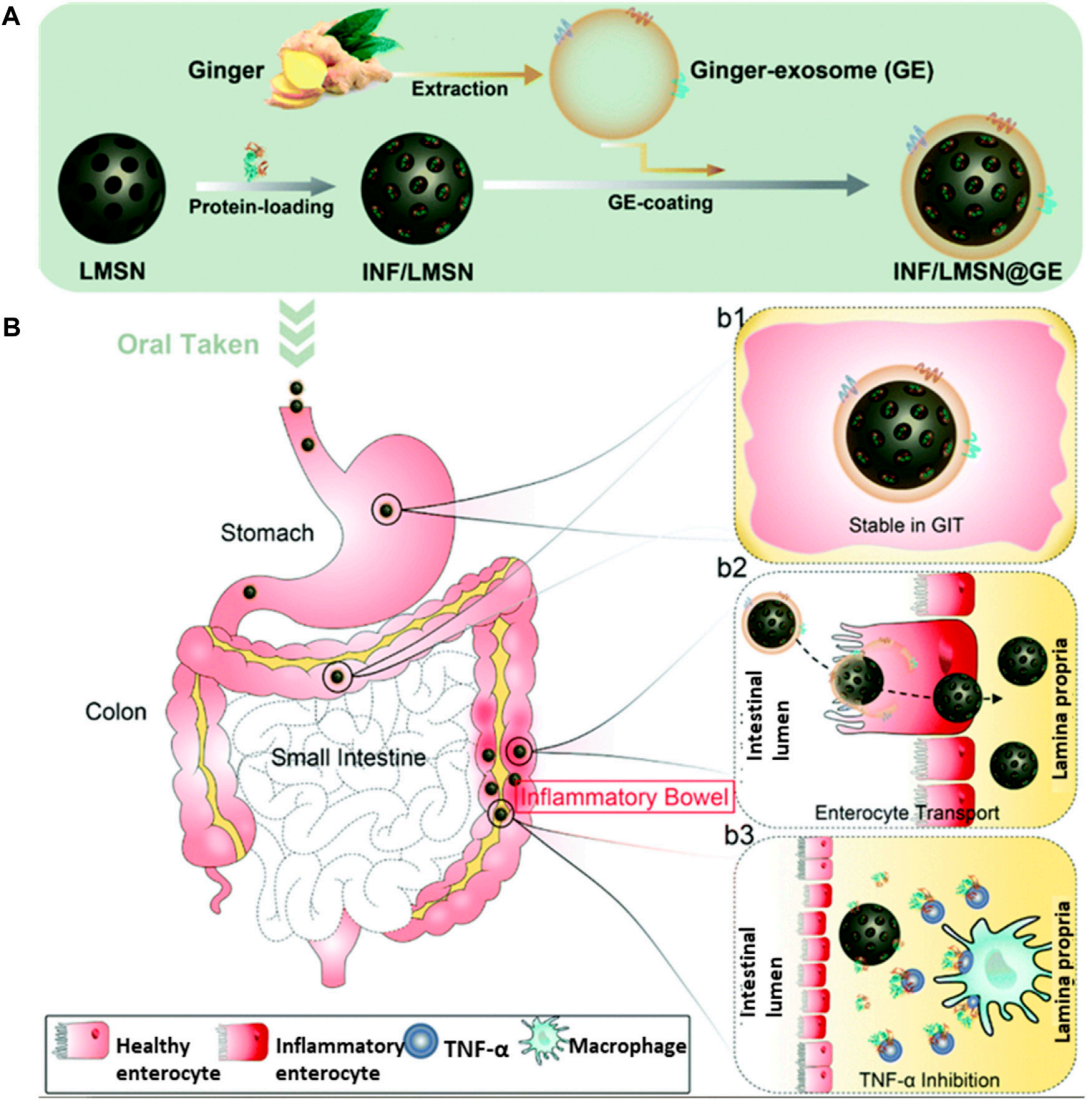
**(A)** An illustration showing the steps in preparing the biomimetic core-shell system. **(B)** Pathways of GIT delivery of IFN-loaded core-shell particle-labeled IFN/LMSN@GE, where LMSN represents large mesoporous silicon nanoparticles and GE represents ginger-derived EVs. Steps involved in the stability of the intact structures while passing through the GI tract and targeting the colonic tissues are shown in b1/b2. Finally, in the colon lamina propria, IFN is released more slowly and inhibits TNF-alpha, as shown in b3 and adapted from Mao et al. (2021). Copyright 2021 Royal Society of Chemistry. GE: ginger EVs; LMSN: large mesoporous silicon nanoparticle; IFN/LMSN: antibody INF-loaded LMSN; LMSN@GE: GE-coated LMSN; IFN/LMSN@GE: antibody INF-loaded LMSN@GE.

Mao *et al.* took advantage of this and designed a nanocarrier based on ginger-derived EVs. However, EVs face the challenges of poor loading of the therapeutic carrier, and to overcome this challenge, they have tried to develop a hybrid system based on an inorganic inner frame made up of silica nanoparticles and biomimetic EV carriers. This hybrid system helped to load the anti-TNF-α antibodies with a loading efficiency of approximately 61.3 wt%, and the outer membrane helped to target the colon tissue specifically. The TNF-loaded hybrid system significantly improved IBD compared with the i.v. of TNF in a colitis mouse model (Figure 6) (Mao et al., 2021).

**FIGURE 7:**
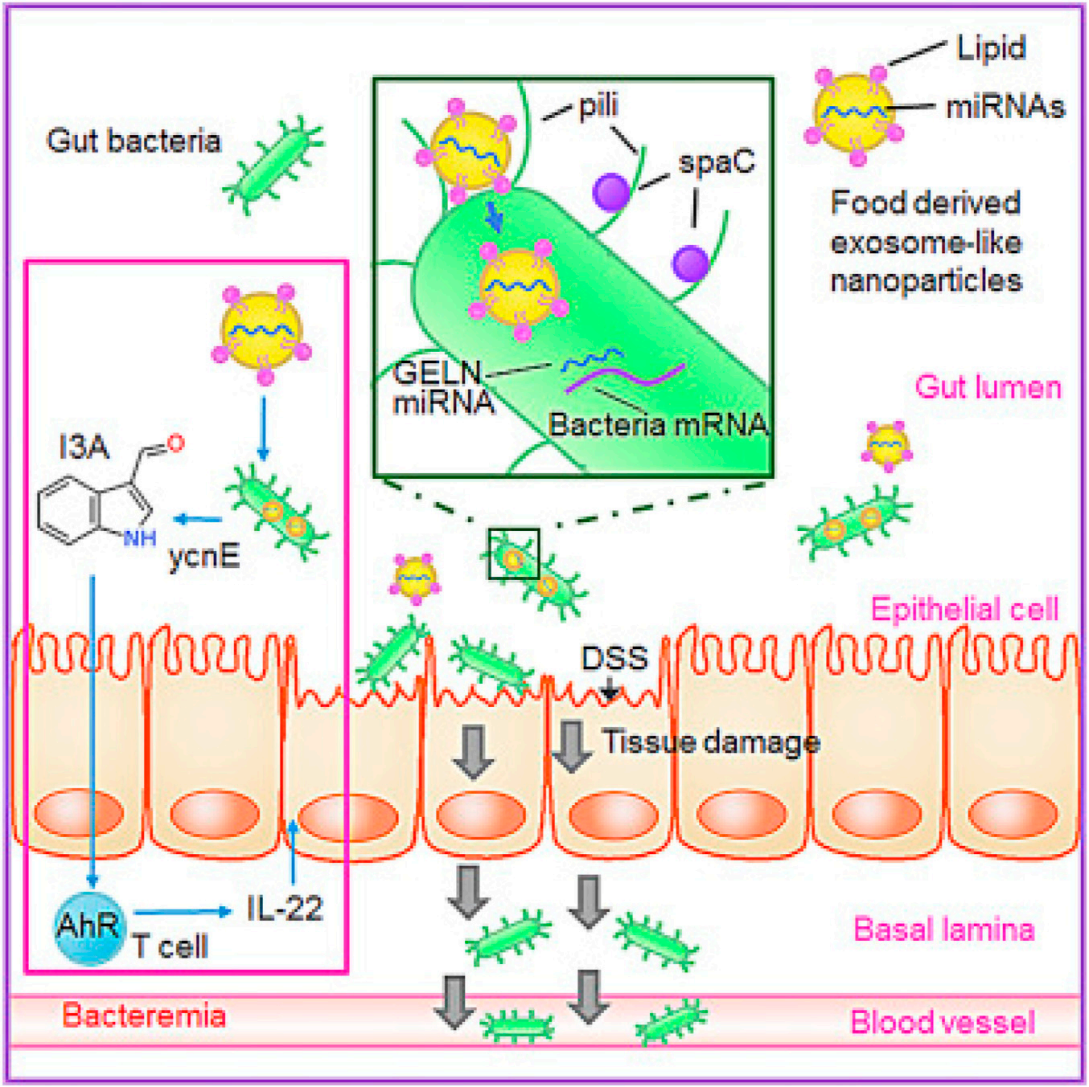
Ginger-derived ELNVs contain RNAs that can help change the gut microbiome’s composition and physiology at the gut site. GELNs are selectively taken up by Lactobacillaceae family bacteria, and microRNAs can target genes in LGG. The above image has been reproduced with permission from Ref Teng et al. (2018). Copyright 2018 Elsevier. GDENs: ginger-derived ELNVs; DSS: dextran sulfate sodium; I3A: indole-3-carboxaldehyde.

Ginger-derived EVs not only help to target specific cells but also help to show anti-inflammatory effects. This effect could be due to reduced proinflammatory cytokines and enhanced anti-inflammatory cytokines. This study clearly shows the potential of differently derived plant-based EVs in various diseases by combining their natural ability and targeting potential. Apart from the anti-inflammatory ability of ginger, derived particles could be used to target gut bacteria selectively. As shown by Teng *et al.* in their proof-of-concept study, ginger-derived EV-like nanovesicles (GDENs) can help to selectively target the specific gut bacteria *Lactobacillus* rhamnosus (LGG) (Teng et al., 2018). As shown in Figure 7, the gut microbiota can be targeted with the help of miRNAs present in ginger-derived ELNVs and can be used to strengthen gut barrier function.

In another study, garlic EV-like nanovesicles (GaELNs), an abundant source, were investigated to reverse high-fat diet (HFD)-induced obesity in mice. Excitingly, GaELNs were administered orally and significantly reduced brain inflammation, as obesity induces chronic inflammation. Moreover, HFD-fed mice showed drastic improvements in memory function, glucose tolerance, and insulin sensitivity in GaELN-treated mice (Sundaram et al., 2022).

**FIGURE 8:**
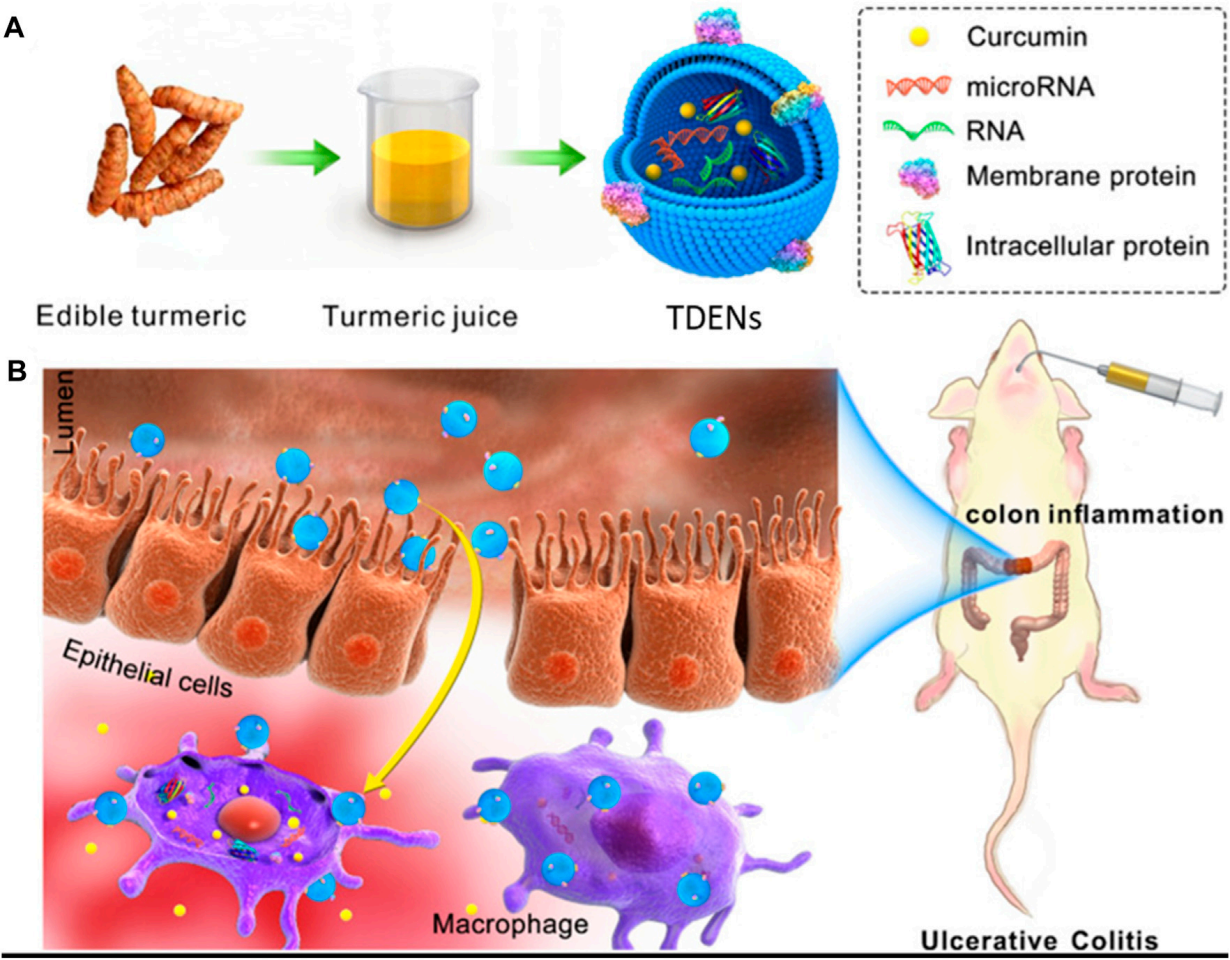
**(A)** Schematic presentation of turmeric-derived EV-like nanovesicle (TDEN) isolation and **(B)** oral administration to target the colon and treat ulcerative colitis (UC). Reproduced with permission from Ref Liu et al. (2022). Copyright 2022 Springer Nature. TDENs: turmeric-derived ELNVs.

Liu *et al.* demonstrated another strategy to treat ulcerative colitis (UC) by using turmeric-derived ELNVs (TDENs), an abundant and easy-to-scale source for biomedical applications. TDENs were isolated and purified by ultracentrifugation and showed physiochemical properties similar to those of EVs. TDENs containing the bioactive ingredient curcumin were investigated as therapeutic agents and administered by oral gavage in an LPS-induced colitis mouse model. This study indicated that TDENs, as site-specific targeting agents, were preferably targeted to inflamed colon tissues and internalized by epithelial cells and macrophages. As a proof of concept of a therapeutic agent, TDENs successfully alleviated inflamed colon tissues and promoted wound healing by regulating TNF-α, IL-6, IL-1β, and HO-1 (Figure 8) (Liu et al., 2022). Furthermore, Yang *et al.* extracted EV-like nanoparticles from Curcumae Rhizoma (CELNs) to develop a promising delivery system. Here, ultrasonication loaded Astragalus components (AC) into CELNs (AC-CELNs). In Caco-2 cell models, AC-CELNs displayed better cellular uptake and transmembrane transport capability. Furthermore, pharmacokinetics demonstrated that the AC-CELNs enhanced the practical components’ absorption within AC *in vivo*. In this investigation, AC and AC-CELNs demonstrated certain degrees of tumor cell activity inhibition after treatment for 6 h incubation. Therefore, CELNs could be considered a promising delivery, and AC-CELNs could potentiate synergistic antitumor activity (Yang et al., 2023).

### 4.2 Oral delivery of fruit-derived EVs

Another interesting class of EVs can be derived from fruits and shows great promise as a delivery carrier with an intrinsic ability to heal specific diseases, targetability toward particular cells or tissues, and anti-inflammatory properties. EVs or EV-like particles from grapefruit were used as carriers to treat gut colitis (Ju et al., 2013). Additionally, grapefruit-derived EVs increased the activation of nuclear factor (erythroid-derived 2)-like-2 (Nrf2) in macrophages. Collectively, these recent findings open up new avenues in the future for delivering macromolecules to gut parts in a protected manner, and they can be used to modulate the gut microbiota depending on their selectivity toward important bacterial families. More research is needed to test the enormous potential of plants, fruit-derived EVs, and ELNV carriers in many disease conditions.

Interestingly, Ju *et al.* used grape EV-like nanovesicles (GELNs) for oral administration in mice. This study revealed that GELNs could penetrate the GI tract and cause significant induction of Lgr5hi intestinal stem cells through the Wnt/β-catenin pathway. Oral administration of GELNs provided markedly improved protection from DSS-induced colitis (Ju et al., 2013).

Alternatives such as acerola, a popular health food-derived EV, have been explored for oral administration of miRNA. miRNA-encapsulating acerola EVs efficiently suppressed the target gene in the GI tract and liver after oral administration and could be a potential carrier for future drug delivery systems (DDSs) (Umezu et al., 2021).

### 4.3 EVs such as nanovesicles from other plant sources

Plant parts such as seeds or bark show great promise as delivery carriers with an intrinsic ability to heal specific diseases, targetability toward particular cells or tissues, and anti-inflammatory properties.

#### 4.3.1 Stem

Sriwastva *et al.* investigated whether EV-like nanovesicles from mulberry bark (MBELNs) could protect against colitis in a mouse model. In this study, MBELNs were used to treat DSS-induced colitis and resulted in significant improvement against gut-related inflammatory disease via the aryl hydrocarbon receptor (AhR)/constitutive photomorphogenic homolog subunit 8 (COPS8) pathway (Sriwastva et al., 2022).

#### 4.3.2 Leaves

ELNVs from green tea leaves decreased reactive oxygen species production, inhibited proinflammatory cytokine expression, and increased IL-10 production by macrophages. Through oral administration of ELNVs, inflammatory bowel disease could be inhibited, colonic barriers could be restored, and the abundance of gut microbiota diversity could be improved, contributing to the prevention or amelioration of inflammatory bowel disease, colon cancer, and colitis (Zu et al., 2021). Importantly, Catharanthus roseus (L.) Don leaf-derived EV-like nanovesicles (CLDENs) were isolated to investigate their immunostimulatory effect in a mouse model. The hydrodynamic sizes and surface charge were 75.51 ± 10.19 nm and −21.8 mV, respectively. In addition, CLDENs displayed excellent stability in GI track environments, such as severe pH and enzymatic degradation. Interestingly, CLDENs showed immune organ targeting ability and altered immunosuppression induced by cyclophosphamide through the TNF-α/NF-κB/PU.1 axis after intraperitoneal injection. This study also demonstrated the biodistribution of CLDENs by oral gavage, which resulted in significant organ accumulation. CLDENs can be mass-produced for industrial use and may have immunomodulatory properties (Ou et al., 2023).

#### 4.3.3 Flowers

Tea leaves have profound health benefits and have impacted medical fields for decades. Hence, tea flowers have not been instantiated for medical applications and are discarded on farms. Chen and his group explored these waste materials to investigate their bioactive potential. Therefore, they investigated tea flower-derived EV-like nanovesicles (TFENs) in metastatic breast cancer, causing 11.6% of total cancer deaths. Excitingly, TFENs were revealed to produce high amounts of ROS, which leads to cell apoptosis via mitochondrial damage and the cell arrest pathway administered by I.V. or oral route. The results showed that both MCF-7 and 4T1 cells had mitochondrial damage, as evidenced by elevated levels of caspase-3 expression after TFEN treatment. On the other hand, the expression levels of BCL-2 were reduced by TFEN treatment compared to nontreatment. Moreover, TFEN treatment decreased two critical cell cycle regulatory proteins, CYCLIN A and CYCLIN B, resulting in cell death. According to the study, TFENs preferentially accumulate in tumor sites, which led to the discovery of potential targeting agents to tumor sites. Additionally, the overall therapeutic outcomes revealed the great success of TFEN treatment via both I.V. and oral routes (Liu et al., 2022).

This finding unraveled a route for developing safe and cost-effective strategies for using plant-derived ELNVs in a therapeutic delivery system. We have summarized information on EVs used for the oral delivery of bovine milk and plant sources (Table 1).

**TABLE 1:** Summary of EVs and ELNVs used for therapeutic aspects via oral delivery.

Sources		Amount of dose	Study outcomes	Ref.
Milk	Cow	4 mg/kg, twice a week	Tumor xenografts from nude mice were significantly ablated (60%; p=0.001) by EVs carried with PAC	Agrawal et al. (2017a)
	Bovine and Porcine	Milk feeding on days 0, 3, 6, and 12	Neither bovine nor porcine milk EVs significantly changed miRNA (miR-2284×, miR-2291, miR-7134, miR-1343, miR-500, and miR-223) levels in piglet serum	Lin et al. (2020)
	Bovine	80 mg/kg, multiple-dose	ExoCUR resulted in significant improvement of antiproliferative and higher anti-inflammatory activity against human lung and breast cancer cells compared to free CUR and PBS as a control	Aqil et al. (2017b)
			ExoCUR inhibited significant changes in tumor growth by ∼61% against the cervical tumor xenograft model	
		8 mg/kg, WFA and 25 mg/kg EV protein, three doses a week	In cell study and lung tumor xenograft model, chemotherapy, including WFA, Anthos, and Cur, was found to enhance therapeutic efficacy in EV-loaded chemotherapy drugs, such as PAC and DOC	Munagala et al. (2016)
			Folate conjugated EV showed improvement in tumor targetability and reduction of tumor growth compared to free drugs	
		1.25 mg/mL of exosomal protein, one time	To evaluate the integrity of RNA loaded into EV under harsh conditions such as GI	Shandilya et al. (2017)
		12 μg of exosomal protein, one time	To evaluate the integrity of RNA loaded into EV under harsh conditions such as GI	Warren et al. (2021)
			Enhanced cellular intake and suppressed GFP gene expression	
		50 UI/kg, single dose	To evaluate the proficient delivery of insulin into EV	Wu et al. (2022)
			Showed significantly higher mucus penetration	
			This resulted in an excellent hypoglycemic effect on type 1 diabetic mice compared to subcutaneous injection of insulin	
		8 mg/kg, single dose	Showed significantly higher mucus penetration	Qu et al. (2022)
			Decreased bacterial loads in the large intestine compared to LZD and AMG	
			The amount of Lactobacillus and Lactococcus genus in the chicken cecum was increased after treatment of AExo	
		6 mg/kg Anthos, 4 mg/kg PAC, and 60 mg/kg EV protein, three doses per week	ExoAnthos improved antiproliferative activity against ovarian cancer cells (OVCA433) than Anthos and vehicle alone	Aqil et al. (2017a)
			The synergistic effect of antitumor activity improved significantly compared to ExoPAC and ExoAnthos treatment in A2780 tumor xenografts	
	Bovine colostrum	6 mg/kg, three times a week	FA-ExoPAC showed a significant reduction in tumor xenografts (50%) compared to PAC alone	Kandimalla et al. (2021)
Root	Ginger	3.3 nmol of siRNA, twice a day	Effectively targeted to colon tissues. Showed significant downregulation of CD98 expression	Zhang et al. (2017)
		0.3 mg/mouse of GDNPs everyday	Reduced acute colitis, improved intestinal tissues, and protection from chronic colitis compared to PBS	Zhang et al. (2016a)
		50 mg/mouse of GDN/day	Accurately targeted to the liver site	Zhuang et al. (2015)
			Demonstrated improvement of alcohol-induced liver disease than no treatment	
		Not mentioned	Evaluated absorption behavior of GDENs in GI in rats	Man et al. (2021)
		10 mg/kg per day	Silica nanoparticles coated with GDENs and loaded with anti-TNF-α antibodies	Mao et al. (2021)
			Specifically, targeted colon tissue and improved IBD	
		0.5 mg/kg per day	Specifically, targeted gut site	Teng et al. (2018)
			IL-22-dependent pathway showed a significant reduction of mouse colitis	
	Ginger, Grape, Carrot	2 mg/mouse per day	Ginger-derived EVs preferably induced heme oxygenase-1 (HO-1) and anti-inflammatory cytokines such as IL-10, white grape- and carrot-derived EVs promoted activation of nuclear factors such as erythroid-derived 2	Mu et al. (2014)
	Garlic	10^10^ particles every day	GaELNs significantly reduced brain inflammation	Sundaram et al. (2022)
			Showed drastic improvement of memory function as well as reduction of obesity in HFD-fed mice	
	Turmeric	3 mg/dose every day	Preferably targeted to inflamed colon tissues and internalized by epithelial cells and macrophages	Liu et al. (2022)
			TDENs successfully alleviated inflamed colon tissues and promoted wound healing	
	Curcumae Rhizoma	1.875 g/kg, single dose	Displayed better cellular uptake and transmembrane transport capability	Yang et al. (2023)
			AC-CELNs enhanced the practical components' absorption within AC in vivo	
			AC-CELNs demonstrated certain degrees of tumor cell activity inhibition	
Fruit	Acerola	10 mg/kg, single dose	Downregulation of the miRNA’s target gene and significant cytoplasmic localization in cervical cancer cell line (SiHa) by treatment of miR-340 loaded EVs.	Umezu et al. (2021)
			Showed gene expressing the effect of small RNA in GI and liver after one day	
	Grape	1 mg/mouse, Single dose	GELNs markedly improved intestinal stem cell proliferation and DDS-induced colitis compared to PBS as a control	Ju et al. (2013)
	Lemon juice	Not mentioned	Lemon-derived EV showed in vitro antineoplastic activity on human cancer cell lines A549, SW480, and LAMA84 compared to PBS as control.	Raimondo et al. (2015)
			Suppressed tumor growth in vivo of Chronic myeloid leukemia (CML) xenograft model.	
			Showed anticancer activity by stimulating a TNF-related apoptosis-inducing ligand (TRAIL)-mediated apoptotic mechanism	

## 5 Engineered ELNVs for oral delivery

Engineered edible plants can deliver siRNAs that inhibit malignant growth in a productive, protected, harmless, and chemopreventive manner at a minimal cost to humans. The loading efficiency of cargo molecules was lower than that of positively charged molecules. However, electroporation does not integrate larger molecules, such as mRNA and DNA, as it does short RNAs (Lamichhane et al., 2015). ELNV production can be exploited in other ways to load RNA, including passive loading, which involves transfecting the source cells with desired cargo through viral or nonviral nano vectors (Ohno et al., 2013). These techniques can potentially overcome the present procedural constraints associated with RNA loading onto ELNVs, broadening the scope of gene delivery. Plant bioengineering to generate miRNAs of any preferred sequence is a remarkable technique, and generating therapeutic miRNAs using edible plants holds great potential in various applications (Zhang et al., 2016b; Munir et al., 2020). Available plant-derived nanocarriers are efficient delivery vehicles for synthetic RNA in a stable and active form (Dad et al., 2021). A colon cancer mouse model was used to evaluate the advantages of plant-based miRNAs (Iravani and Varma, 2019). The oral gavage of a mixed drink of three plants anticancer inhibitor miRNA mimics with a methyl functional group at position 20 of 30 nucleotides reduced the growth of colon cancer. In addition, the serum levels of plant miR159 were conversely associated with malignant neoplasia progression and cancer incidence in breast cancer. Most of the studies identified multiple copies of miR159 in EVs. Preliminary *in vitro* studies have shown that the production of miR159, representing a sequence of 3′ untranslated responses (3’ UTR) of intercept factor 7 mRNA, can reduce the development of malignant cells. Oral administration of miR159 mimetics generally limited the growth of mammary tumors in mouse xenografts (Iravani and Varma, 2019).

Various hidden systems facilitating the permeability of exogenous miRNAs in the gut include.

A) In the epithelium of the digestive tract, there is endless retention of RNA-encompassing compounded miRNAs into absorbed EVs. While some entangled endosomes age and degrade into lysosomes, some can fuse with endosomes and undergo transcytosis, thereby carrying macromolecules to the other side of the biological barrier.
B) Gastrointestinal tissues containing lymphocytes may receive RNA carriers delivered to macrophages by microfold cells (M-cells) in the Peyer’s patch of the digestive tract. Tissue immune cells are crucial in distributing RNA-containing complexes throughout the body.
C) Enhanced biological function may result from the uptake of plant miRNAs bound within exosome-like NPs that macrophages or intestinal stem cells take up.
D) These unprotected plant miRNAs can be transported transiently owing to their low stability but can enter the cytoplasm, possibly through transmembrane miRNA transporters or receptor-mediated endocytosis.
E) Treatment of gastrointestinal diseases using conventional drugs and alteration of digestive permeability due to hunger and stress can accelerate the uptake of unfamiliar miRNAs.

## 6 Uptake mechanism of EVs

**FIGURE 9:**
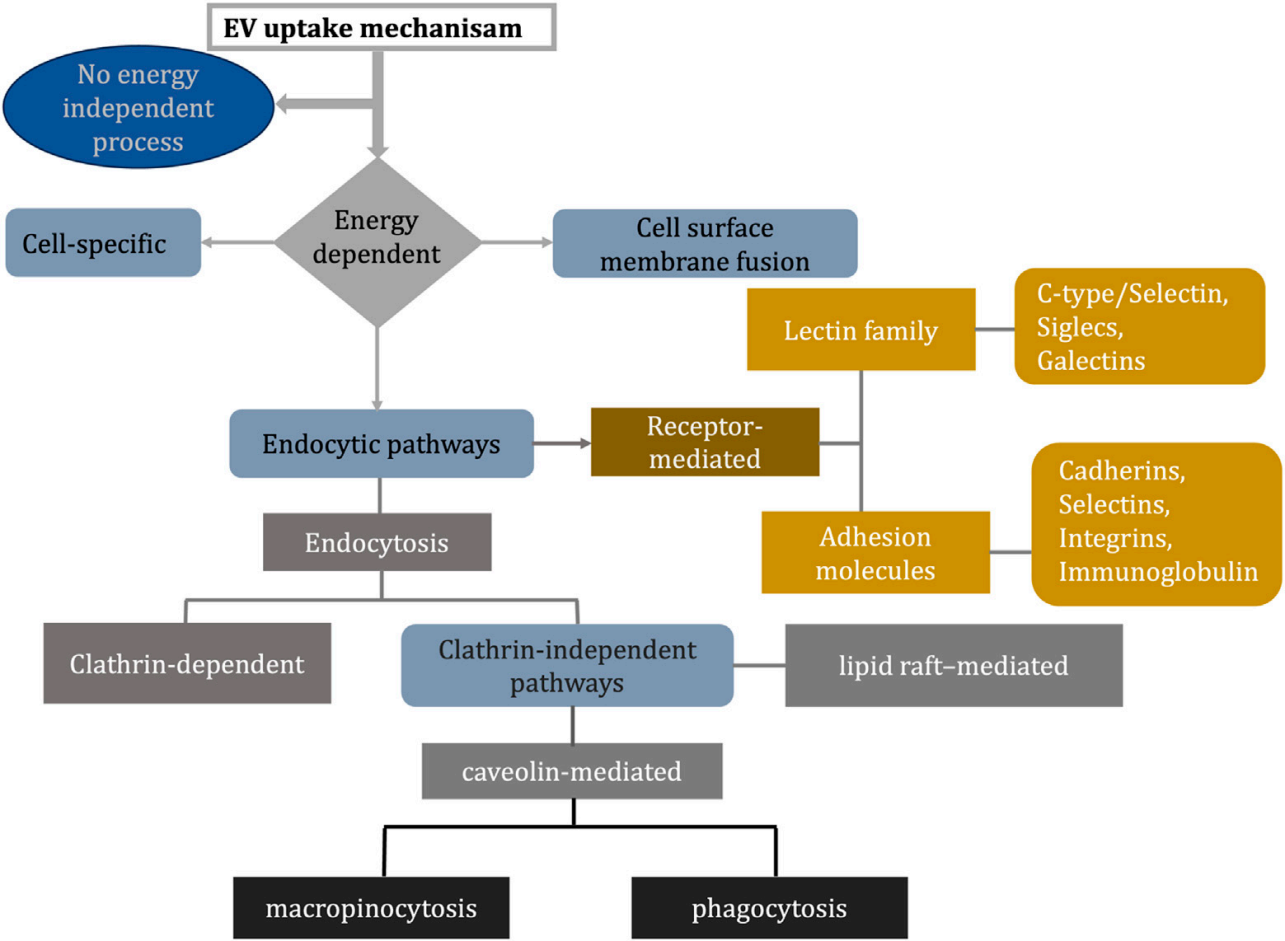
Flowchart explains different ways of EV uptake mechanism (Mulcahy et al., 2014; Mylvaganam et al., 2021; Detampel et al., 2022).

However, the mechanism of internalization of EVs into cells remains an area of great debate in the literature. Various mechanisms can lead to EV uptake, including clathrin-mediated endocytosis (CME), phagocytosis, macropinocytosis, and membrane fusions between plasma and endosomes. We discussed some of the proteins involved in EV uptake and illustrated the different uptake mechanisms as a flowchart in Figure 9 (Mulcahy et al., 2014).

Tetraspanins: Tetraspanins belong to a family of membrane proteins crucial for adhesion and motility, activation, and proliferation of cells. The presence of tetraspanins on EV surfaces suggests they may play a role in EV function. There are three well-established markers of EVs: CD63, CD9, and CD81. Microdomains enriched in tetraspanin (TEM) are found in raft-like structures in the plasma membrane and are composed of tetraspanin, adhesion molecules, and transmembrane receptor proteins. The hypothesis that TEMs play a role in EV-cell binding is supported by their involvement in various processes, including vesicular and cellular fusion (Mulcahy et al., 2014).

Integrins and immunoglobulins: Integrins and immunoglobulins play an essential role in the interaction between vesicles and cells due to their role in the immune response. These proteins perform A range of functions, including adhesion between cells, signaling between cells, migration of leukocytes across endothelial cells, and presenting antigens. It has been suggested that they may also facilitate the uptake of EVs (Mulcahy et al., 2014).

A role for proteoglycans: Proteins that contain carbohydrates are proteoglycans. Proteins with sulfated glycosaminoglycan polysaccharides are called heparin sulfate proteoglycans (HSPGs). In order to enter cells, HSPGs are used by various complexes, including viral particles and lipoproteins (Mulcahy et al., 2014).

Lectins: DC-SIGNs are classified as C-type lectin receptors (can internalize glycoprotein ligands) capable of triggering phagocytic entry for virus and bacterial particles. Breast milk EVs and epithelial cells contain the MUC1 protein, a potential ligand (Mulcahy et al., 2014).

Endocytosis: Studies suggest that endocytosis usually occurs for EVs taking up endosomal compartments. A rapid uptake of EVs is observed, with detection as early as 15 min after EVs are introduced (Mulcahy et al., 2014).

Clathrin-mediated endocytosis: Cells internalize molecules by assembling transmembrane receptors and their ligands into clathrin-coated vesicles. By strategically deforming the membrane, the clathrin-coated vesicles form avesicular buds, mature, and pinch off. As the clathrin is uncoated, the intracellular vesicle joins the endosome, where its contents are deposited (Mulcahy et al., 2014).

Caveolin-dependent endocytosis: Cellular endocytosis is heavily studied, but evidence suggests eukaryotes have multiple clathrin-independent pathways. An example of such a mechanism is caveolin-dependent endocytosis (CDE). Like clathrin-coated pits, caveolae can be internalized into cells and act as cave-like invaginations in the plasma membrane. In addition to cholesterol, sphingolipids, and caveolins, caveolae are sub-domains of rafts of glycolipids of the plasma membrane so that CDE can be sensitive to cholesterol-depleting agents such as filipin and methyl-b-cyclodextrin (MbCD). An invagination of a membrane by caveolae requires caveolin-1, a protein that is present in clusters within the invaginations. The formation of caveolin-rich rafts in the plasma membrane is mediated by caveolin oligomerization (facilitated by caveolin oligomerization domains) (Mulcahy et al., 2014).

Micropinocytosis: In micropinocytosis, membrane ruffles are pushed into the intracellular compartment through invagination of the membrane. The membrane ruffles and extracellular fluid around the membrane are carried in the vesicles. Fusion of the membrane protrusions with themselves or the plasma membrane causes this extracellular fluid area to be internalized entirely, as the plasma membrane’s ruffled extensions extend from the cell surface to cover an area of extracellular fluid. Like phagocytosis, the mechanism does not require direct contact with the internalized material. Actin, cholesterol, and rac1 are all required for the mechanism to work and Na/Exchanger activity (Mulcahy et al., 2014).

Phagocytosis: Externalizing opsonized particles like bacteria and fragments of apoptotic cells is a process of phagocytosis. A macrophage can perform this function. During phagocytosis, receptors become involved and progressive invaginations form around the material being internalized, with or without enveloping membrane extensions (as required in micropinocytosis). In general, larger particles are internalized by phagocytosis. EVs, however, may be internalized by phagocytosis since particles as small as 85 nm have been internalized by phagocytosis (Mulcahy et al., 2014).

Involvement of lipid rafts: Membranes contain microdomains of lipid rafts that have distinct phospholipid compositions. Sphingolipids like sphingomyelin and protein receptors are abundant in them. In addition to regulating membrane fluidity and interfering with protein trafficking, they assemble signaling molecules. A lipid raft consists of highly ordered, tightly packed components, which are less fluid in the plasma membrane than the surrounding bilayer. Usually, lipid rafts provide cholesterol, which is needed in clathrin-independent endocytosis. Lipid raft domains may be involved in the internalization of EVs by cells (Mulcahy et al., 2014).

Cell surface membrane fusion: The EV membrane can fuse directly with the cell’s plasma membrane. When two initially distinct membranes merge in an aqueous environment, they form a lipid bilayer. A hemi-fusion stalk with fused outer leaves is formed when lipid bilayers come into direct contact and the outer leaflets merge. A fusion pore is formed due to stalk expansion following hemi-fusion of the diaphragm bilayer. Thus, a consistent structure results from the mixture of the two hydrophobic cores. This process involves several protein families, including SNAREs, Rab proteins, and Sec1/Munc-18 related proteins (SM-proteins) (Mulcahy et al., 2014).

Cell-specific EV uptake: EV uptake is currently a subject of debate within the field, as it is unclear whether it is a cell-specific phenomenon. Others claim vesicular uptake is a precise process that can only occur between cells with the right combination of ligands and receptors. The observed discrepancies may be due to heterogeneity in donor or recipient cells, EVs, experimental setup, or context (Mulcahy et al., 2014).

### Targeting strategies for EVs and ELNVs

Since milk EVs possess several unique characteristics, they may be used to diagnose and treat disease. Nevertheless, ongoing research into targeted modifications and utilization of milk EVs faces some limitations that require further exploration. A folate-modified milk EV is more likely to accumulate in tumor tissues when endocytosis is induced by folic acid (Munagala et al., 2016). Munagala *et al.* demonstrated the modification of milk EVs loaded with withaferin A (WFA) in the context of breast cancer treatment. As a result of folic acid modification, the drug became more bioavailable orally, which resulted in a significant decrease in tumor growth. (Munagala et al., 2016).

A study by Kandimalla et al., in 2021 modified milk EVs with paclitaxel (PAC) and folic acid (FA) (Munagala et al., 2016). It was found that oral administration of FA-Exo-PAC significantly inhibited xenogeneic subcutaneous tumors. Furthermore, FA-Exo-PAC was more effective than traditional intravenous injections of PAC in an orthotopic lung cancer model. Wu et al. obtained CD44-specific ligands using hyaluronic acid (HA), developed in 2020, to encapsulate doxorubicin (Dox) combined with azithromycin, DSPE-PEG2000-HA and *in vitro* experiments demonstrated that HA-Exo-Dox selectively bound to CD44-overexpressing cancer cells, specifically to breast cancer cells and lung cancer cells. As a result, cancer cell survival rates decreased significantly compared to treatment with free Dox (Li et al., 2020). Moreover, Li et al. prepared tumor-specific microRNA-204-5p analogs by coating milk EVs with hyaluronic acid (HA-mExos). An enhanced antitumor effect of HA-mExo-miR204 was observed *in vitro* and *in vivo* when targeted specifically towards CD44-positive cancer cells (Li et al., 2022). Modifying milk EVs with galactose and N-acetyl galactose reduced EV accumulation in the liver and pancreas and broadened their distribution throughout the body (Sukreet et al., 2019).

**FIGURE 10:**
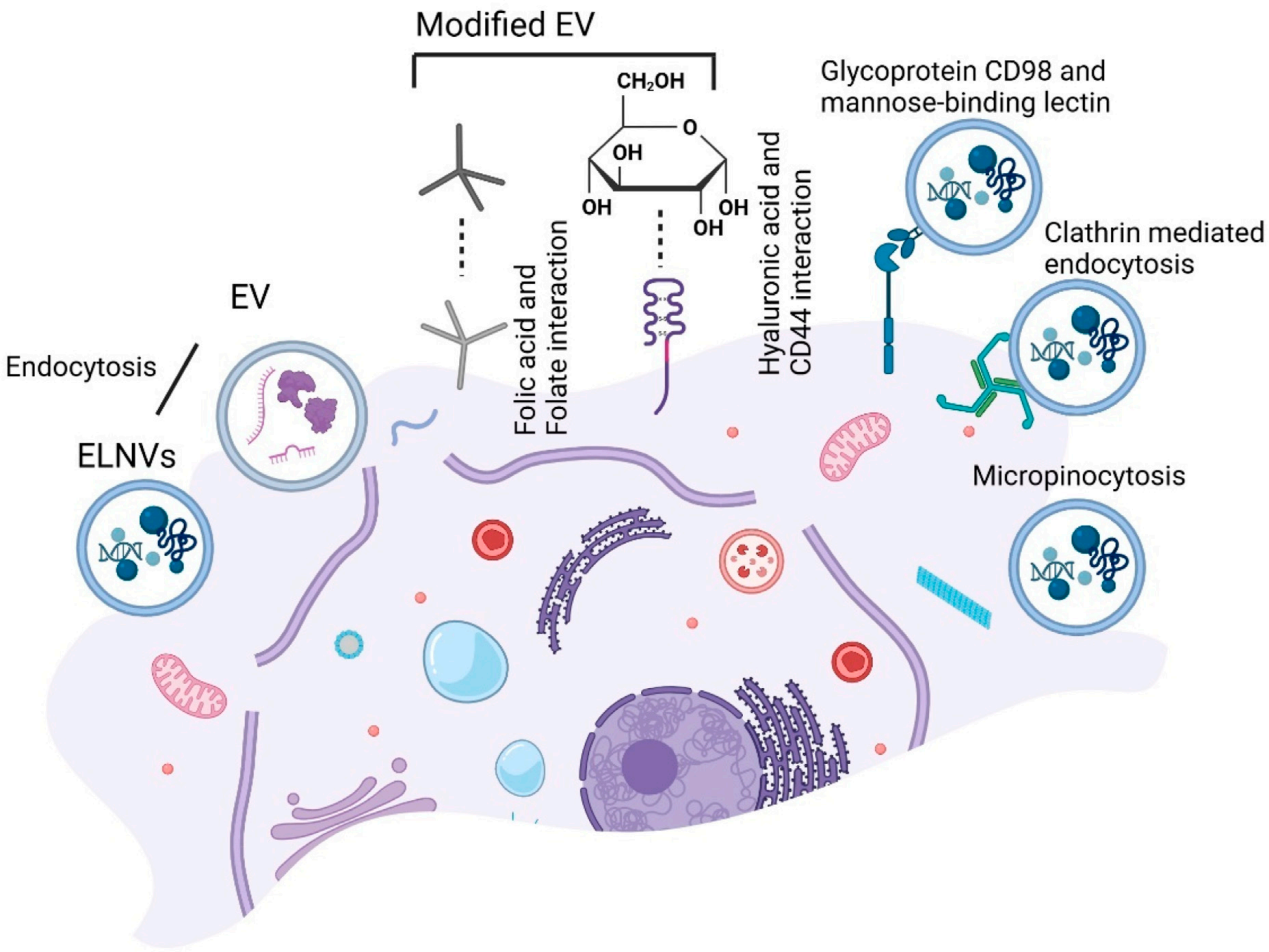
Schematic presentation of the targeting strategy for extracellular vesicles (EV) endocytosis uptake. Folate-modified EV endocytosis by folic acid and Hyaluronic acid (HA) modified EV endocytosis by CD44. While exosome-like nanovesicles (ELNVs) endocytosis and further with glycoprotein CD98 and a mannose-binding lectin, clathrin-mediated and micropinocytosis.

A study demonstrated that garlic-derived nanovesicles can interact with liver cells via the transmembrane glycoprotein heterodimer CD98 and a mannose-binding lectin (Song et al., 2020). Unlike grape-derived EVs, grape EVs require micropinocytosis and clathrin-dependent pathways to enter macrophages (Wang et al., 2014). We have illustrated the targeting strategies for modified milk EVs and ELNVs in Figure 10.

## 7 Clinical perspectives on EVs and ELNVs: advantages and limitations

### Advantages

Similar in structure to cell membranes, EVs are natural carriers for small molecules to be delivered directly to cells. As a result of their small size, they can cross physiological barriers, including the blood-brain barrier, and avoid macrophage phagocytosis, allowing them to circulate in the body for extended periods and avoid rapid degradation (Adriano et al., 2021). Several studies have confirmed that EVs are effective drug delivery systems because of their superior circulation capacity and bioavailability (Admyre et al., 2007).

Milk EVs have distinct advantages as a drug delivery system compared to other EVs. In addition to being biocompatible and biodegradable, they exhibit low toxicity and immunogenicity (Somiya et al., 2018). The inherent anticancer properties of milk EVs make them practical for modulating inflammation. Secondly, drug solubility is enhanced by contact with milk EVs since they have a larger surface area. By resisting acid attacks, low pH environments, and pepsin, these EVs protect the drug from degradation in the gastrointestinal tract (Izumi et al., 2012). Through milk EVs, cancer drugs are transported to the oral cavity, circumventing the effects of the gastrointestinal tract’s first pass, thereby improving their bioavailability. According to studies, milk EVs protect macrophages from cytotoxic effects induced by chemotherapy drugs, such as cisplatin (Matic et al., 2020). By encapsulating anticancer drugs within milk EVs, the toxicity of the drugs is reduced as compared to the administration of pure drugs. Milk EVs are also highly stable in the blood, enabling them to travel long distances both physiologically and pathologically in the body (Munagala et al., 2016). Depending on the specific targeting functions or desired response characteristics, they can be further modified or designed to acquire additional desired characteristics.

In addition to their remarkable biocompatibility and tissue penetration abilities and overcoming physiochemical barriers, plant-derived EV-like nanoparticles (PELNs) exert anti-inflammatory and antitumor effects directly at the lesion site due to their physicochemical stability. A natural PELN is highly biocompatible, stable, dispersed *in vivo*, has a prolonged half-life, and attains cellular internalization. As they can load various substances, including nucleic acids, recombinant proteins, and small molecules, they are a cost-effective and promising drug delivery platform. As a result of their large-scale production from abundant plant resources, PELNs are attractive as delivery devices for drug delivery (Wiklander et al., 2019).

### Limitations

Although milk EVs have potential applications, they are limited in their widespread use. It is important to note that laboratory requirements for isolating milk EVs differ substantially from those in industrial production. Separation and extraction technologies available today have inherent limitations, and their suitability for industrial use remains imperfect. In addition, there is no standard or strict means of purifying EVs for industrial usage. A second challenge is delivering drugs into milk EVs and modifying them in a targeted manner. Several factors must be addressed, including the drug’s nature and the development of methods for stabilizing its loading into milk EVs (Tian et al., 2023).

**FIGURE 11:**
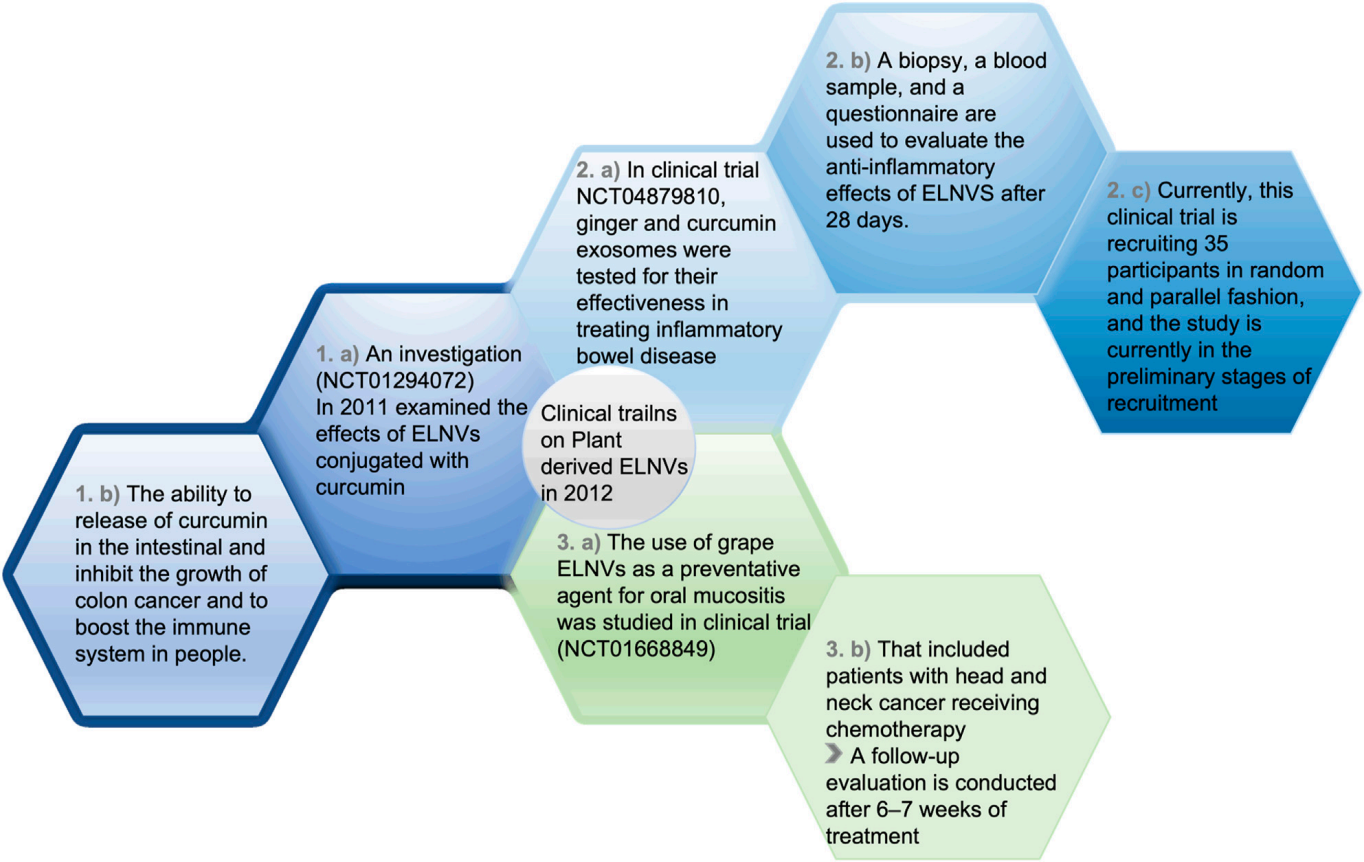
Clinical studies and their progress on ELNVs.

The EVs from plants, which contain diverse biomolecules, may pose unknown risks and side effects. Despite its simplicity, convenience, and relative safety, oral administration has the disadvantage that first-pass liver elimination significantly reduces the concentration and dose of the drug in the systemic circulation. The efficiency of treatment is also negatively impacted by slow absorption rates. Additionally, intravenous administration is an option, but a unified plan for separating and extracting EVs is currently lacking. Depending on the method used, EVs can vary in content and purity, resulting in heterogeneity and affecting experimental outcomes. Seasonal and geographical factors further complicate the study of plant EVs since some plant species only grow in specific regions or seasons, adding complexity to this field (Mu et al., 2023). Additionally, EVs and ELNVs offer safety, availability, and cost-effectiveness when treating diseases. Plants, milk, and other cells are believed to contain EVs and ELNVs that protect the intestinal barrier and maintain the gut microbiota. Due to their excellent biodistribution and inherent biocompatibility, EVs and ELNVs effectively deliver drugs orally; however, targeting specific target sites remains challenging. Shortly, EVs and ELNVs may be used to treat diseases, but further research must be conducted to determine their mechanisms, dosage, and adverse effects before they are approved for clinical application (Figure 11).

## 8 Conclusion and future directions

EVs and ELNVs have potential applications for drug delivery. In recent years, scientists have been increasingly interested in unraveling the composition and therapeutic potential of EVs and ELNVs. The complexity of EVs and ELNVs (scale, purity, cost, consistency, and standardization) and their diversity in structures and sizes has been demonstrated in numerous studies. They may have distinct characteristics depending on which species EVs and ELNVs are obtained from. It is also important to note that no uniform and standardized separation and purification method exists.

In contrast to basic laboratory research, industrial research has entirely different requirements than basic laboratory research. Hence, the separation method varies depending on the purity and quantity of EVs. A technical problem must be solved first to translate EV drug research into clinical practice. It is one of the main drawbacks to expanding the EV market. It is required to optimize the purification methods, improve the homogeneity of EV preparations, and standardize the separation technology before entering for the marketing. Natural compounds are delivered to cell targets using EVs and ELNVs, which have wide biocompatibility, low toxicity, low immunogenicity, and a lower allergic response and are incapable of crossing the placenta, reducing their cross-contamination of a growing fetus. Evidence shows that EVs and nanovesicles containing EVs have potential as drug delivery systems. EVs and EV-like nanovesicles are still in their infancy as therapeutic agents because of the difficulties in extraction and purification. Despite this, existing studies suggest that EVs and EV-like nanovesicles may offer promising therapeutic benefits and be worth intensively examining.
